# The frequency and precipitating factors for breakthrough seizures among patients with epilepsy in Uganda

**DOI:** 10.1186/1471-2377-13-182

**Published:** 2013-11-21

**Authors:** Martin Kaddumukasa, Mark Kaddumukasa, Steven Matovu, Elly Katabira

**Affiliations:** 1 Department of Medicine, College of Health Sciences, Makerere University, P. O. Box 7062, Kampala, Uganda

**Keywords:** Breakthrough seizures, Epilepsy, Precipitating factors

## Abstract

**Background:**

Epilepsy is one of the major brain disorders worldwide. Breakthrough seizures carry a heavy burden of epilepsy, with increased morbidity and risk of premature mortality. Several factors have been suggested to precipitate break through seizures but these have not been studied in our setting. The study sought to determine the prevalence of breakthrough seizures, as well as precipitating factors in adults with epilepsy attending Mulago hospital.

**Methods:**

This study was conducted in Mulago Hospital, using a cross sectional study design between August and December 2009. Subjects with epilepsy and had been receiving anti-epileptics treatment for at least 6 months prior to the study were consecutively enrolled.

**Results:**

A total of 256 patients with epilepsy were recruited. Prevalence of breakthrough seizures among epilepsy patients attending Mulago hospital was 75.3%. Factors found to be significantly associated with breakthrough seizures were non compliance to anti-epileptic therapy (p < 0.0001); duration of treatment (p < 0.0001); infections (p < *0.044*) and menses among female study participants (p < 0.0001). The level of education, sleep deprivation, alcohol and substance abuse, and flickering lights were not associated with breakthrough seizures.

**Conclusions:**

Breakthrough seizures are high in Mulago National referral hospital, with drug non-compliance the commonest cause. The attending physicians need to identify precipitating factors among patients attending Mulago hospital and have them addressed appropriately during patient care.

## Background

Epilepsy is one of the commonest serious neurological disorders worldwide, affecting an estimated 50 million people. In Uganda, epilepsy is the commonest neurological disorder with an estimated prevalence of 2–5 persons per 1000 people in the country. It has been reported that the number of new epilepsy cases in Uganda in one year were 156 among 100,000 people [1]. Epilepsy may be secondary to the adverse neurological sequelae of viral, bacterial and other parasitic infections (malaria and onchocerciasis) during and beyond childhood [2,3]. Epilepsy has been found to have a high preponderance in onchocerchiasis endemic areas with rates as high as 15–20 cases per 1000 people in Kabarole and Nebbi districts [4]. Approximately 50% of all patients with epilepsy report having seizures of varying frequency and severity despite antiepileptic drug (AED) treatment [5,6]. Continued seizures can impact negatively on work, family, and social life [7,8].

Whereas, the course of epilepsy may vary, it has been reported to display a fluctuating course [9], with up to 30% of patients with epilepsy continuing to have seizures, despite trying a range of AEDs [10]. The relationship between seizures and other external factors is still less clear. Breakthrough seizures are seizures that occur in a person who had previously good consistent control of epilepsy documented in the medical records and then gets another seizure. The International League against Epilepsy considers breakthrough seizures as evidence of inadequate control and hence treatment failure after excluding poor treatment compliance and planned dose reductions [11].

Breakthrough seizures may result from forgotten prescriptions, under dosing, and use of recreation drugs. Other factors that have been proposed include watching television, playing video games, sleep deprivation, exertion and emotional stress [12,13]. In general, seizures that occur under these circumstances are still considered as evidence of inadequate seizure control and hence treatment failure. The treatment with anti-epileptic drugs aims to reduce the number and the severity of seizures to a minimum and at most to none. It is therefore important to identify the various modifiable factors that may affect seizure control once patients are taking AEDs. Stannaway et al., in a London Hospital found that 31% of seizures were precipitated by non-adherence to medication [14].

Despite shouldering the biggest burden of epilepsy, no studies have been done in our setting to determine the burden of breakthrough seizures in Uganda. It was therefore important to determine the prevalence of breakthrough seizures and the possible precipitating modifiable factors among patients with epilepsy in Uganda.

Our study aimed at determining the frequency and modifiable precipitating factors of breakthrough seizures among known patients with epilepsy with prior seizure control. The information gained will provide a basis for standards of patient care and management of epilepsy in our setting.

## Methods

This was a cross sectional study carried out at Mulago National referral and teaching hospital in Kampala, Uganda. Between August and December 2009, two hundred sixty seven (267) subjects attending the neurology and mental health clinics were consecutively enrolled into the study. The inclusion criteria for enrolment into the study were; a) adults with confirmed epilepsy, b) epileptic subjects previously well controlled receiving anti-epileptic drugs (AEDs) therapy for more than 6 months, c) written consent to participate in the study. Participants with neurological impairments such as aphasia were excluded. Epilepsy was defined using the proposed International League Against Epilepsy (ILAE) criteria [15]. Seizure-precipitating factors were defined as those circumstances that precede the onset of an epileptic attack and were considered by both patient and neurologist to be a possible explanation why the seizure happened when it did, and not earlier or later [16]. We assessed the dose-adherence by asking patients two simple questions: if they missed taking any anti –epileptic pills and the number of doses they had missed over the past 7 days. Dose adherence was considered optimal if patients reported ‘no’ to both questions, and suboptimal if they answered ‘yes’ to any question.

Two study sites at Mulago were used to screen for study recruitment; the neurology out-patient clinic and mental health outpatient clinic. These clinics serve as a secondary and tertiary referral center for patients with epilepsy in district surrounding the hospital.

In our study the diagnosis of epilepsy was based on history and clinical assessment and using the ILAE definition for epilepsy [15].

Using a pre-tested standardized questionnaire and review of medical records, we assessed demographic characteristics.

Informed consent was sought from individuals who met the inclusion criteria. By interviewing the patient or parent of the subject using a pre-tested questionnaire, information was obtained on demographic characteristics, behavioral factors, medical history and clinical data. Information on potential seizure precipitants, such as lack of adherence to medication, menstruation, fever and alcohol were collected. A detailed clinical examination was performed and those suspected to have breakthrough seizures were sent to the laboratory for attendant investigations such as blood slide examination for malaria parasites, electrolytes etc. Malaria was defined as fever (axillary temperature more or equal to 37.5°C) plus any *P. falciparum* asexual parasites/μl. Using an operational definition we defined pneumonia when a subject presented with fever, cough, breathlessness, chest pain and auscultatory crepitations in the lung fields. All subjects with breakthrough seizures received anti-epileptic therapy according to the national treatment policy for epilepsy. The study was approved by the Makerere University Faculty of Medicine Research and Ethics Committee, the Mulago hospital review board and the UNCST.

### Statistical analysis

Data was analyzed by STATA statistical software (version 9). We estimated the prevalence as the ratio of cases with breakthrough seizures to the population enrolled into this study.

To determine factors associated with breakthrough seizures, we first performed univariate analysis. We estimated prevalence risk ratios (PRRs) with logistic regression and adjusted for age, sex, marital status, education level, drug type, duration of anti-epileptic therapy, number of drug doses missed and hours slept at night. We tabulated the prevalence risk ratios with 95% confidence intervals. Variables with *P* values <0.25 upon univariate analysis and clinically relevant variables which were determined by the authors, these included age, marital status, educational level, number of doses missed, duration of therapy, hours slept a night and menstruation periods among female study participants were used in the logistic model. We used the prevalence risk ratios instead of odds ratio because high prevalence rates of breakthrough seizures.

## Results

Two hundred and fifty six (256) participants attending the Mulago hospital neurology and mental health outpatient department, were enrolled into the study of which 145 (56.7%) were male. The mean age among the study participants was 27 years (SD ± 11.8 years). See Table 1 for baseline characteristics of the study population.

**Table 1:** Characteristics of patients enrolled in the study

**Characteristics**	**Number of respondents, N**	**Percentage**
Overall	256	100%
**Age (years)**		
Mean (SD)	27 (11.8)	
*Age group*		
18-20	97	37.9
21-29	80	31.2
30-44	56	21.9
45+	23	9.0
**Sex**		
Female	111	43.3
Male	145	56.7
**Marital status**		
Never married/single	195	76.2
Married	45	17.6
Divorced/separated/widowed	16	6.2
**Occupation**		
Non-cash employment	89	34.9
Cash employment	70	27.5
Student	69	27.0
Unemployed	27	10.6
**Level of education**		
None	17	6.6
Primary (P1-P7)	101	39.5
Secondary (S1-S6)	102	39.8
Tertiary	36	14.1

Of the 256 subjects enrolled in this study, one hundred and ninety three study participants (75.4%) had breakthrough seizures. The participants aged between 18 to 20 years had biggest burden of breakthrough seizures at 80.4%. The prevalence of breakthrough seizures reduced with an increase in age. Participants aged 30–44 years had the least prevalence of breakthrough seizures at 69.6%, while 73.9% of those aged more than 45 years had breakthrough seizures. However there was no significant association of breakthrough seizures and age. Thirty (27%) of the 111 female study participants reported that the seizures were worse during the menstruation period. Eleven point seven percent of the study participants had ongoing infections (malaria and pneumonia), ninety percent (27/30) of the infections occurred among those with breakthrough seizures. Malaria was leading infection, followed by pneumonias and diarrhea respectively.

Males had a higher percentage of breakthrough seizures (57.5%), however, this was not significant, p- value – 0.581, (PRR – 0.96, 95% CI 0.84, 1.11). Participants aged 18 to 20 years had most breakthrough seizures (40.4%) while study participants above 45 years had the least occurrence 8.8%. (See Table 2, showing the prevalence of seizures, unadjusted and adjusted Prevalence Risk Ratios (PRR) for breakthrough seizures among the study participants).

**Table 2:** Prevalence of Seizure, unadjusted and adjusted Prevalence Risk Ratios (PRR) for breakthrough seizures

	**Number with seizure n/N**	**Percentage with seizure (%)**	**Unadjusted PRR 95% CI**	**p-value**	Adjusted PRR**, 95% CI	**p-value**
**Sex**						
Female	82/111	73.9	0.96 (0.83,1.11)		0.96 (0.84,1.11)	
Male	111/145	76.6	1.0	0.618	1.0	0.581
**Age group**						
18-20	78/97	80.4	1.0		1.0	
21-29	59/80	73.8	0.92 (0.78, 1.08)	0.301	0.91 (0.76,1.08)	0.266
30-44	39/56	69.6	0.87 (0.71,1.06)	0.157	0.86 (0.68, 1.08)	0.181
45+	17/23	73.9	0.92 (0.71,1.19)	0.529	0.92 (0.65,1.30)	0.633
**Marital status**						
Never married/single	148/195	75.9	1.0		1.0	
Married	35/45	77.8	1.02 (0.86,1.22)	0.784	1.06 (0.85,1.31)	0.602
Divorced/separated/widowed	10/16	62.5	0.82 (0.56, 1.21)	0.327	0.87 (0.56,1.34)	0.531
**Level of education**						
None	13/17	76.5	1.0		1.0	
Primary (P1-P7)	77/101	76.2	1.0 (0.75,1.38)	0.983	0.98 (0.73,1.30)	0.864
Secondary (S1-S6)	77/102	75.5	0.99 (0.74,1.31)	0.930	0.97 (0.73,1.30)	0.842
Tertiary	26/36	72.2	0.94 (0.68, 1.32)	0.737	0.98 (0.70,1.37)	0.897
**Duration on Treatment**						
<1 years	30/32	93.8	1.0		1.0	
**1-5**	**109/150**	**72.7**	***0.78 (0.68,0.89)***	***<0.000***	***0.79 (0.68, 0.91)***	***0.001***
**5+**	**54/74**	**73.0**	***0.78 (0.66,0.92)***	***0.004***	***0.82 (0.68,0.98)***	***0.025***
**Number of drugs taken**						
One drug	64/83	77.1	1.0		1.0	
Two drugs	94/124	75.8	0.98 (0.84,1.15)	0.828	0.98 (0.85,1.15)	0.839
Three drugs	35/49	71.4	0.93 (0.75,1.15)	0.481	0.93 (0.76,1.15)	0.525
**Doses missed**						
None	136/187	72.7	1.0		1.0	
1-3 doses	23/32	71.9	0.99 (0.78,1.25)	0.921	1.04 (0.82,1.30)	0.764
**> 3 doses**	**34/37**	**91.9**	***1.26 (1.11,1.44)***	***<0.0001***	***1.33 (1.16,1.52)***	***<0.000***
**Hours slept (hours)**						
<=6	18/26	69.2	1.0		1.0	
7-9	109/145	75.2	1.09 (0.83,1.43)	0.555	1.11(0.83,1.49)	0.467
10+	66/85	77.7	1.12 (0.85,1.49)	0.424	1.18 (0.87,1.59)	0.283

**Adjusted for age, sex, marital status, education level, drug type, duration of anti-epileptic therapy, number of drug doses missed and hours slept at night.

N- total number of study participants, n – number of subjects with seizures.

Study participants who had been on treatment with anti-epileptic therapy for more than one year but less than five and those more than five years were associated with breakthrough seizures, (p-value 0.001, PRR – 0.79 95% CI 0.68, 0.91) and (p-value 0.025, PRR – 82, 95% CI 0.68 – 0.98) respectively. The odds of study subjects who missed more than three doses of anti-epileptic drugs in the preceding week of enrolment was 1.33 times higher for breakthrough seizures compared to those missed less than three doses. However, there was no association between breakthrough seizures and the number of anti-epileptic drugs taken. The hours slept prior to the occurrence of a seizure, smoking, use of alcohol and level of education were not associated to breakthrough seizures. In this study, flickering lights and watching television was not associated with breakthrough seizures, p-value of 0.176 for unadjusted PRR (95% CI) of 1.12 (0.96-1.26) while 0.187 for the adjusted PRR. There was a significant association between the prevalence of breakthrough seizures and duration of treatment. Study participants who had been receiving anti-epileptic drugs for more than one year had reduced breakthrough seizures with p-values of 0.0001 and 0.004 respectively (see Table 3).

**Table 3:** Proportion with seizures, unadjusted and adjusted Prevalence Risk Ratios (PRR) for breakthrough seizures

	**Number with seizure/Total**	**Percentage with seizure**	**Unadjusted PRR 95% CI**	**p-value**	Adjusted PRR,** 95% CI	**p-value**
**Flickering lights *Watching TV***						
Yes	104/144	72.2	1.0		1.0	
No	89/112	79.5	1.10 (0.96,1.26)	0.176	1.34 (0.87,2.06)	0.187
**Duration of watching TV**						
<1 hour	39/58	67.2	1.0		1.0	
2 hours	54/72	75.0	1.12 (0.89,1.40)	0.340	1.21 (0.96,1.52)	0.105
>4 hours	12/15	80.0	1.19 (0.87,1.62)	0.274	1.67 (0.83,1.64)	0.378
**Alcohol use**						
Yes	13/17	76.5	1.0		1.0	
No	180/239	75.3	0.98 (0.75,1.30)	0.913	0.78 (0.58,1.05)	0.096
**Knowledge of disease**						
Yes	88/113	77.9	1.0		1.0	
No	105/143	73.4	0.94 (0.82,1.08)	0.408	0.84 (0.69,1.02)	0.084
**Menses**						
Seizures worse during menses	27/30	90.0	1.0		1.0	
Not worse in menses	53/79	67.1	***0.75 (0.61,0.91)***	***0.003***	***0.75 (0.62,0.91)***	***0.004***
**Infections**	27/30	90.0	***0.83 (0.10,1.12)***	***0.003***	***0.75 (0.62, 091)***	***0.004***

**Adjusted for age, sex, marital status, education level, drug type, duration of anti-epileptic therapy, number of drug doses missed and hours slept at night.

In multivariate model, parameters with p values less than 0.25 were considered. The shorter duration of treatment was significantly associated with breakthrough seizures. Study participants receiving anti-epileptic treatment for one to five years and those more than five years had a lower prevalence risk of breakthrough seizures. The unadjusted PRR was 0.78, 95% CI (0.68, 0.89), p –value 0.000 and 0.78, 95% CI (0.66, 0.92), p-value 0.004 respectively. The adjusted PRR was 0.79, 95% CI (0.68, 0.91), p-value 0.001 and 0.82, 95% CI (0.68, 0.98), p-value 0.025 respectively. Non compliance to anti-epileptic drug treatment was significantly associated with break through seizures, adjusted PRR = *1.33 (95% CI, 1.16 – 1.52,* p < 0.001). Menstruation periods among the female study participants was also significantly associated with breakthrough seizures with an adjusted PRR = 0.75 *(95*% *C, 0.62 – 0.91,* p = 0.004*)*. Co-current infections during the clinic visit due to malaria and bacterial pneumonias were significantly associated with breakthrough seizures with an adjusted PRR = 0.*85 (*95% CI 0.*10 – 0.112,* p = 0.039).

Non compliance and infections, and menses among the female study participants were the most common precipitants at 46.6%, 16.6% and 14.5% respectively. Noise (1%) and breast feeding (0.5%) were among the least reported precipitants.

## Discussion

In our study we found an overall age adjusted prevalence of breakthrough seizures of 75.3%, which is higher than in other studies [1,12,17]. This high rate of breakthrough seizures may be multi-factorial and is concerning. Some of these factors may be health system related while others are patient related. Addressing these factors will go a long way in helping control the high rate of breakthrough seizures in our clinic. The Ministry of Health is responsible for the provision of adequate, good quality anti-epileptic drugs but this has been lacking at several occasions. Mulago hospital being the national referral hospital with a neurology specialist outpatient clinic might also be receiving patients with refractory epilepsy who have been referred for neurologist specialist care. Addressing these factors may help reduce breakthrough seizures among patients in our setting. Patient tailored education to address lack of knowledge regarding the problem of drug discontinuation, spiritual illusional thoughts about epilepsy, frustration and mental impairment. We need to work with the government to address issues of drug cost and income disparity, unemployment, lack of uniform availability of drugs in local market. Uniform availability of cheaper antiepileptic drugs with adequate information and communication regarding the disease and uplifting of socio-economic status would play a big role in reducing breakthrough seizures.

There was a slightly lower prevalence of breakthrough seizures in females (73.9%) compared to male (76.6%) epileptic subjects. This may be due to a better drug compliance and patient health seeking behavior. Female patients are generally more complaint to therapy and seek health care better when compared to men [18-20]. The precipitating factors for seizures were drug non-compliance (46.6%), infections such as malaria, bacterial pneumonia and urosepsis (16.6%), and menstruation (14.5%), sleep deprivation (11.4%) among the study participants. These precipitating factors may interact with the environmental factors in a complex fashion and may increase the risk of breakthrough seizures [21]. These findings differ considerably from previous studies done in Norway, Denmark and USA with non-compliance at 3.7%, infections 5.1%, menstruation 3.3% and emotional stress 20.9% [12]. The high percentage of non-compliance (46%) in Mulago hospital as a cause of breakthrough seizures may be attributed to anti-epileptic drug stock outs and the patients or care-takers may be out of pocket to maintain a sustained availability of anti-epileptic drugs among the participants. In this study, non compliance occurred in the low income strata and 80% of subjects who missed their doses reported lack of funds as a reason for non compliance. In a UK study which is more consistent with this study found that 31% of seizures may be attributable to medication non compliance [14]. Non compliance may be an important factor we need to address to reduce this high prevalence of breakthrough seizures at Mulago hospital. Some studies have shown that 30 – 50% of the patients with epilepsy are reported to be noncompliant to the extent of interfering with optimal treatment and seizure control [22,23].

We also found a significant relationship between the duration of treatment on antiepileptic drugs and the occurrence of breakthrough seizures. Study participants who were on treatment for more than one year had the lowest risk for seizures. (p = 0.004, CI 0.68-0.91). This might be explained by regular routine physician-patient drug discussions which reinforces good drug compliance. These discussions help in identifying possible causes of irregular medication which can be corrected. These results are similar to earlier findings that have been reported by Joyce et al. [24].

Poor adherence to the epilepsy treatment regimen is likely one important contributor to continued seizures. In this study, 91.9% of the participants who missed more than three doses in the week preceding enrolment were more likely to develop seizures. This is similar to other studies which have reported seizures after missing a dose of medication and the association of non adherence with higher seizure frequency in adults with epilepsy [24-26]. Furthermore, non adherence may affect clinical decision-making resulting from the underestimation of AED efficacy and tolerability and overestimation of dosing requirement for optimal efficacy. This further increases the costs of care especially in resource limited settings like Uganda. However, the limiting factor of non - adherence in our study would have been recall bias, this might have been understated. Other techniques like electronic drug bottle monitors would have been preferred to document adherence. Therefore, regular assessment and supporting interventions to promote anti-epileptic drug adherence should be maintained throughout the course of therapy. Identifying effective strategies which can used to ensure adherence to therapy would help reduce this high prevalence of non-compliance.

Infections had significant associations with breakthrough seizures (p =0.044), these included malaria and pneumonias. The mechanism through which infections cause seizures is still poorly understood, however it’s thought to cause vasculature damage and compromise the integrity of the blood brain barrier [27-29]. Infections may also increase emotional stress among subjects with epilepsy which is another precipitant of seizures. Infections cause a robust inflammatory response, cytokines, and impaired homeostasis and increase in vascular permeability [30,31]. Infections are associated with febrile seizures which may predispose to epilepsy. Prolonged or focal febrile seizures have been associated with the development of limbic (temporal lobe) epilepsy [32].

Menstruation was strongly associated with breakthrough seizures among the female study participants. Similar findings have been reported elsewhere about 78% of women developed seizures in the peri - ovulatory phase or during the ovulatory phase of the menstrual cycle ( p <0.001) [33]. Herzog et al. has described three standards of seizure worsening related to different periods of the menstrual cycle [33]. This is due to the hormonal changes and a decrease in progesterone levels which is anti-epileptic. Seizures are more likely to occur near the time of menstrual flow because of progesterone withdrawal and with the estrogen surge at ovulation. These seizures occurring during menstruation can occur at any time of the menstrual period and are severe during the anovulatory cycles because the high ratio of estrogen to progesterone [34].

## Conclusion

The occurrence of breakthrough seizures may be multi-factorial in our setting. More attention is needed for issues related to gender like seizures occurring during menses especially when providing care to female patients with epilepsy. Efforts should be made to address the improvement of the economic status of care-givers or patients as this will less drug non-compliance as well as transport costs to attend scheduled clinic visits due to out of pocket. We need to improve and strengthen the Ugandan health system in regards to provision of adequate good quality anti-epileptic drugs for all patients with epilepsy. This will reduce on missed doses and sub optimal treatment subsequently reducing the high prevalence of breakthrough seizures at Mulago. It is important to identify and appropriately address known precipitants such as infections (malaria and pneumonias) and emphasizing better patient health seeking behavior for their attendant problems. These coupled with routine regular doctor patient discussions will help to control breakthrough seizures among patients with epilepsy attending Mulago hospital.

## Competing interests

None directly related to this study for all authors.

## Author’s contributions

MK participated in the coordination of the study and data collection. SM participated in design of study and data collection. MK participated in design of the study and drafted the manuscript. EK conceived the idea of the study, and participated in its design and helped to draft the manuscript. All authors read and approved the final manuscript.

## Pre-publication history

The pre-publication history for this paper can be accessed here:

http://www.biomedcentral.com/1471-2377/13/182/prepub
